# Customized Integrating-Sphere System for Absolute Color Measurement of Silk Cocoon with Corrugated Microstructure

**DOI:** 10.3390/s23249778

**Published:** 2023-12-12

**Authors:** Riaz Muhammad, Seok-Ho Lee, Kay-Thwe Htun, Ezekiel Edward Nettey-Oppong, Ahmed Ali, Hyun-Woo Jeong, Young-Seek Seok, Seong-Wan Kim, Seung-Ho Choi

**Affiliations:** 1 Department of Biomedical Engineering, Yonsei University, Wonju 26493, Republic of Korea; riaz@yonsei.ac.kr (R.M.); josephlee@yonsei.ac.kr (S.-H.L.); kaythwetun90@gmail.com (K.-T.H.); ezekieledward@yonsei.ac.kr (E.E.N.-O.); alee@yonsei.ac.kr (A.A.); 2 Department of Integrative Medicine, Major in Digital Healthcare, Yonsei University College of Medicine, Seoul 06229, Republic of Korea; 3 Medical Physics and Biomedical Engineering Lab, Yonsei University College of Medicine, Seoul 03722, Republic of Korea; 4 Department of Electrical Engineering, Sukkur IBA University, Sukkur 65200, Pakistan; 5 Department of Biomedical Engineering, Eulji University, Seongnam 13135, Republic of Korea; hwjeong@eulji.ac.kr; 6 Gangwon-do Agricultural Product Registered Seed Station, Chuncheon 24410, Republic of Korea; air5738@korea.kr; 7 Department of Agricultural Biology, National Institute of Agricultural Sciences, Rural Development Administration, Wanju 55365, Republic of Korea

**Keywords:** absolute color measurement, silk cocoon, integrating sphere, diffuse reflection, color checker, color calibration

## Abstract

Silk fiber, recognized as a versatile bioresource, holds wide-ranging significance in agriculture and the textile industry. During the breeding of silkworms to yield new varieties, optical sensing techniques have been employed to distinguish the colors of silk cocoons, aiming to assess their improved suitability across diverse industries. Despite visual comparison retaining its primary role in differentiating colors among a range of silk fibers, the presence of uneven surface texture leads to color distortion and inconsistent color perception at varying viewing angles. As a result, these distorted and inconsistent visual assessments contribute to unnecessary fiber wastage within the textile industry. To solve these issues, we have devised an optical system employing an integrating sphere to deliver consistent and uniform illumination from all orientations. Utilizing a ColorChecker, we calibrated the RGB values of silk cocoon images taken within the integrating sphere setup. This process accurately extracts the authentic RGB values of the silk cocoons. Our study not only helps in unraveling the intricate color of silk cocoons but also presents a unique approach applicable to various specimens with uneven surface textures.

## 1. Introduction

Silk fiber, a versatile bioresource, finds extensive utility across agriculture, the textile industry, and biomedical engineering [1]. The most used silk is obtained from the cocoons of the larvae of the mulberry silkworm *Bombyx mori*. The history of silk dates to ancient China, where it was first developed and used for clothing and other textiles [2]. With high mechanical strength and excellent optical transparency, silk also functions as a biomaterial in various biological and medical fields [3]. The breeding of silkworms to yield new varieties has been an ongoing process for centuries to improve the quality and quantity of silk produced [4,5]. The breeding of silkworms stands as a crucial element in the creation of valuable textile materials [5]. One of the objectives of silkworm breeding is to improve the quality and quantity of silk production, as well as the health of the silkworms. To achieve this, silkworm breeders need to monitor and measure various traits of the silkworms and their cocoons, such as weight, size, color, texture, and strength. Among these traits, cocoon color is particularly important, as it affects the appearance and value of silk products. During this process, optical sensing techniques have been employed to distinguish the colors of silk cocoons, aiming to assess their improved suitability across diverse industries [6,7,8,9]. A silk cocoon color measurement system can also help silkworm breeders understand the genetic and environmental factors that influence cocoon color, such as temperature, humidity, nutrition, and disease [10,11]. By using this system, silkworm breeders can optimize their breeding conditions and strategies to produce silkworms with desirable cocoon colors [12].

**Figure 1:**
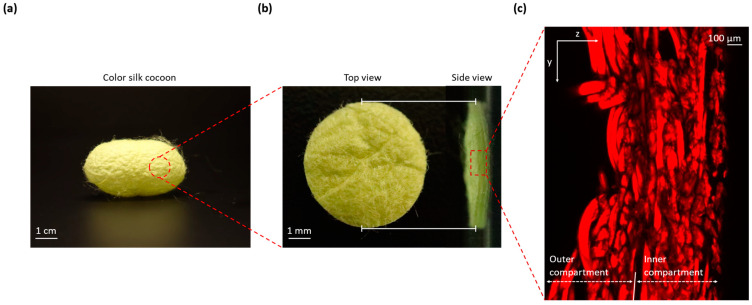
Illustrations of the silk cocoon surface with corrugated microstructure: (**a**) Silk cocoon. Displays a comprehensive photograph capturing the entirety of the silk cocoon. This image offers a clear view of the cocoon’s external surface texture, characterized by the presence of distinct corrugations; (**b**) Cross-section of silk cocoon cut piece. Zooms in on a precisely cut 1 cm diameter section from the silk cocoon. This allows for a closer examination of both the external surface (left) and a cross-sectional view (right), revealing the intricate corrugated microstructure responsible for the cocoon’s unique texture; (**c**) Confocal analysis of silk cocoon cross-section. Presents a confocal image of a cross-section from the silk cocoon, showcasing the detailed and woven nature of the uneven corrugated microstructure of the silk cocoon surface.

The surface texture of silk cocoons exhibits a remarkable and intricate pattern characterized by an uneven and corrugated microstructure [13]. This corrugated microstructure is defined by a series of undulating wrinkles that traverse the surface of the silk cocoon [14]. These wrinkles create variations in height and depth, resulting in a textured landscape reminiscent of undulating hills and valleys. This unique topography can lead to color distortion and inconsistent color perception when viewed from different angles. Figure 1a displays a comprehensive photograph capturing the entirety of the silk cocoon. This image shows a clear view of the cocoon’s external surface texture, characterized by the presence of distinct corrugations. Figure 1b zooms in on a precisely cut 1 cm diameter section from the silk cocoon. This allows for a closer examination of both the external surface (left) and a cross-sectional view (right), revealing the intricate corrugated microstructure responsible for the cocoon’s unique texture. Figure 1c presents a confocal image of cross-sections from the silk cocoon, showcasing the detailed and woven nature of the heterogeneously structured corrugated microstructure of the silk cocoon surface [14].

Several methods are currently employed to discern the colors of silk fibers derived from silk cocoons, facilitating their versatile industry applications. Previous techniques for evaluating the color characteristics of silk cocoons have employed various techniques. Spectrophotometry has been used to analyze the reflected light spectrum from silk fibers to ascertain their color [15], while chemical tests investigate color-related chemical properties [16]. An existing method involved utilizing a Data-color 650 colorimeter to analyze the color index of both gold cocoon silk and gold-like cocoon silk [17]. Another widely employed approach utilized digital cameras to comprehensively examine the color and external features of silk cocoons [8,18,19,20]. A distinct method involved cutting and scanning multiple cocoons using an integrating sphere over a wavelength range of 260 nm to 700 nm with a white cocoon serving as a control [21]. Additionally, researchers have utilized the RGB color component difference as a threshold to discern the color variations in silk cocoons [22]. In another study, visible spectroscopy was employed to evaluate the yellowish index of non-woven silk fabrics according to the CIE 1931 color space [23]. This method involves analyzing the spectral properties of light reflected from the silk fabrics, particularly focusing on the yellowish hue within the color space.

These diverse techniques employed for ascertaining the color of silk cocoons exhibit several limitations, such as the cost and susceptibility to external factors affecting imaging conditions, thereby impeding the attainment of absolute color measurements. The current techniques fall short of providing an absolute depiction of the color of silk cocoons, primarily due to inherent constraints in calibration precision and variations in external environmental conditions during imaging processes.

Moreover, most of these methods assess the color only after extracting the silk fibers from the cocoons. If color could be determined at the silk cocoon level, it would enable the selective breeding of silk cocoons with desired colors, thereby streamlining the extraction process. While visual comparison remains the predominant method for distinguishing differently colored silk cocoons [24], the uneven texture of the silk cocoon, with its corrugated microstructure, creates shadows and color distortion during conventional color measurement methods. Consequently, current visual color discrimination methods result in unnecessary fiber waste in the textile industry and potential experimental errors in medical fields.

**Figure 2:**
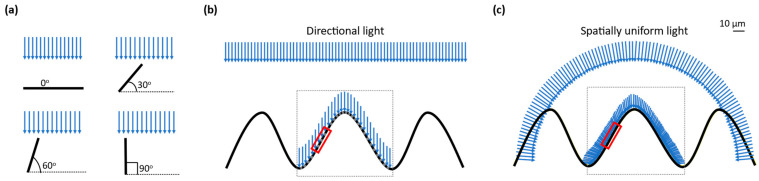
Effects of different lighting conditions on silk cocoon color measurement: (**a**) When directional light is directed onto the uneven surface of a silk cocoon characterized by corrugated microstructures, the resulting luminance distribution (cd/m^2^) varies across the surface, as described by Lambert’s Cosine Law, which relates luminance to the angle (*θ*) between the surface normal and the incident light direction. This effect is visually demonstrated in diagrams depicting angles of incidence at 0°, 30°, 60°, and 90°; (**b**) Luminance variations on textured silk cocoon create shadows and highlights. When directional light is directed onto the textured silk cocoon, it creates uneven luminance (cd/m²) due to shadows and highlights, as shown within the red frame. This hampers the reliability of color measurements; (**c**) Ensuring uniform illumination with an integrating sphere. By diffusing light uniformly in all directions, the integrating sphere ensures consistent and even illumination across the cocoon’s surface (as demonstrated in the red rectangular frame). This minimizes shadows and highlights caused by roughness, leading to enhanced precision and reliability in color measurement.

When a directional light from any light source is used to shine on the uneven surface of a silk cocoon, the brightness (luminance in cd/m^2^) is not the same everywhere on the silk cocoon surface. This happens because the luminance on the uneven surface of the silk cocoon with a corrugated microstructure is proportional to the cosine of the angle between the surface and the light source (Figure 2a). Therefore, when the corrugated microstructures on the surface of the silk cocoon are at an oblique angle to the directional light, the luminance is reduced, resulting in shadows and highlights on the rough areas and making some parts look brighter or darker than others (Figure 2b). This uneven distribution of luminance can make color measurement challenging and less reliable.

Uneven luminance distribution can affect the color measurement by causing variations in the perceived color due to differences in lighting across the sample [25,26]. The uneven luminance distribution can lead to shadowing and highlights, which may affect the perceived color of the silk cocoon. To mitigate this, uniform illumination techniques such as diffuse lighting or multiple light sources can be employed to ensure consistent and accurate color measurement. Thus, uneven luminance distribution can lead to inaccurate color measurements, impacting the quality assessment of silk cocoons. Employing uniform illumination techniques is essential to ensure accurate and reliable color measurement in the evaluation of silk cocoon quality.

A promising solution to this problem is utilizing an integrating sphere. The integrating sphere scatters light in all directions, resulting in a diffused and evenly distributed illumination across the silk cocoon surface (see Figure 2c). Furthermore, this unique illumination system has the advantage of eliminating shadows originating from the uneven surface, as light arriving from multiple directions counteracts any shadow effects. Consequently, the corrugated microstructure of the silk cocoon’s surface appears smoother, enabling precise color extraction through mathematical analysis. This uniform illumination significantly enhances the accuracy and consistency of color measurements.

The relationship between luminance and the angle between the surface and the rays of the light source can be expressed mathematically as:

(1) $$L_{v}=L_{vo}\cos(\theta )$$

where *L_v_* is the luminance on the surface, *L_vo_* is the luminance of the light source, and *θ* is the angle between the surface and the light source. This equation shows that the luminance on the surface decreases as the angle between the surface and the light source increases, resulting in shadows and highlights on the rough areas. The use of an integrating sphere eliminates these shadows and provides a diffused and evenly distributed illumination, resulting in more accurate and consistent color measurements.

To determine the absolute color of silk cocoons with their corrugated microstructures, we developed an optical setup consisting of a customized integrating sphere, a color reference standard (ColorChecker), and a camera. Opting for a digital camera combined with a customized integrating sphere to measure silk cocoon color, as opposed to prior use of fiber spectrometer setups [12,15], was due to the intricate way silk cocoons scatter and reflect light. The reflected light from the surface of silk cocoons is significantly influenced by the surface heterogeneities and measuring geometry. The customized integrating sphere serves to uniformly illuminate the textured surface of silk cocoons, enabling precise capture by the digital camera. Additionally, the high resolution of the camera, versatility, and affordability facilitate the absolute color measurement of the silk cocoons, crucial for understanding unique cocoon traits. Next, we employed the ColorChecker and performed a color calibration using MATLAB R2022b to precisely calibrate the RGB values obtained from the ColorChecker within the integrating sphere. Once calibration was complete, we computed the absolute color RGB values of the silk cocoons using the calibrated functions.

Obtaining the absolute color of silk cocoons is imperative for accurate and reliable analysis in various fields, including textile industry quality control and scientific research. Absolute color measurement ensures a standardized and consistent basis for comparison, enabling researchers and industry professionals to precisely evaluate color variations in silk cocoons. This is particularly crucial in applications where subtle color distinctions may carry significant implications, such as in the assessment of silk quality or the identification of specific characteristics related to cocoon health. Therefore, addressing the limitations of current methods is essential for enhancing the accuracy and robustness of silk cocoon color analysis.

## 2. Materials and Methods

### 2.1. Materials

#### 2.1.1. Integrating Sphere

A custom integrating sphere was used in this study. The primary reason for customization is that the sample should be positioned at the center of the sphere to receive illumination from all directions, including the front and back. However, commercially available integrating spheres are reflection-based, requiring sample measurement at the port’s end. Additionally, absolute calibration, a key part of characterization, demands measurement to be made over a large imaging area. Traditional integrating spheres fail to meet these requirements for extensive imaging and illumination due to limitations in their exit ports relative to the internal area, hindering their effective functioning. To provide large imaging and illumination areas, we have designed a customized integrating sphere where the sample is placed at the center of the sphere. This type of configuration has never been attempted in other integrating sphere products.

**Figure 3:**
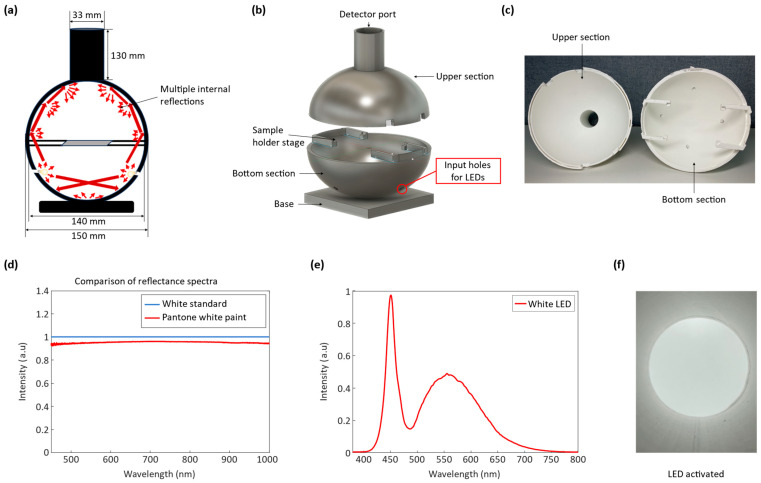
Design and configuration of LED-based customized integrating sphere for creating spatially uniform illumination: (**a**) This illustration serves as a visual guide to the fundamental operation of our customized integrating sphere, thoughtfully designed with a detector mount for precision. Incoming light, characterized by wave-like properties, interacts with the sphere’s inner walls, generating multiple reflections, as illustrated by the dark red arrows, and resulting in uniform irradiance throughout. This spatially integrated illumination minimizes directional bias, providing consistent, evenly distributed light for sample illumination; (**b**) 3D Design of our customized integrating sphere. Designed using Autodesk Fusion 360 (© 2023 Autodesk, Inc., San Francisco, CA, USA). This integrating sphere consists of two sections: an upper section featuring a camera mount and a lower section equipped with supports for holding the sample stage, holes for inserting light-emitting diodes (LEDs), and a base for balancing the integrating sphere; (**c**) Uniform interior coating of a 3D printed integrating sphere with white paint. The cross-sectional view of the 3D-printed integrating sphere, coated with white paint, reveals uniform paint coverage on both sections: the upper section (on the left) and the bottom section (on the right) of the sphere’s interior. This consistent paint application ensures even illumination within the integrating sphere; (**d**) The reflectance spectrum of the white paint used as the coating material inside the integrating sphere shows a close resemblance to the reflectance spectrum of the Spectralon white standard (model USRS-99-010, Labsphere Inc., North Sutton, NH, USA); (**e**) The measured spectrum of a white LED. The spectrum was recorded using a compact CCD spectrometer (model CCS200/M, Thorlabs, Newton, NJ, USA), capable of measuring a wavelength range of 200 nm to 1000 nm; (**f**) The interior of the sphere after closing the upper section and mounting an array of four LEDs in the designated holes in the lower section of the integrating sphere. The LEDs were powered at 2.7 V, and the resulting image captured by the camera shows the sphere interior uniformly illuminated in all directions. The diffuse lighting in the sphere highlights the linearity of the light source, demonstrating the ability of the LED light source to effectively function as an input light source for the integrating sphere.

An integrating sphere, also known as the Ulbricht sphere, is an optical device that is used to spatially integrate radiant flux, resulting in the diffusion or even distribution of light. The integrating sphere is known for its ability to distribute light uniformly from all directions [27,28]. The sphere is a hollow structure with small apertures that serve as entry and exit points for light (see Figure 3a). The inner surface of the sphere is coated with a highly reflective material, ensuring that the walls scatter light diffusely [29,30]. This property enables light to undergo multiple scattering reflections inside the sphere, resulting in a homogenous distribution of light at the sphere’s center. The scattered light further interacts with the integrating spheres’ paint particles, causing additional diffusion and reflection, thereby generating a uniform and diffuse illumination within the sphere. This method is crucial for attaining a uniform light distribution and minimizing directional effects within the integrating sphere. As a result, the integrating sphere provides a neutral and uniformly reflective surface, exhibiting consistent diffuse reflectance across different incident wavelengths [31]. The operation of the integrating sphere is explained through a mathematical equation that describes radiance. The overall expression for radiance, denoted as *L*, associated with a diffusely illuminated surface receiving an input flux, Φ*_i_*, can be formulated as follows:

(2) $$L=\frac{\Phi_{i}R}{\pi A}$$

where *R*, *π,* and *A* are the reflectance, the total projected solid angle originating from the diffuse surface, and the illuminated area, respectively [32]. The radiance equation of an internally illuminated integrating sphere accounts for both the multiple surface reflections and the losses incurred through the port apertures needed for the input flux.

(3) $$L=\frac{\Phi_{i}}{\pi A_{s}}\;\times \;M$$

The radiance equation consists of two parts; the first part matches the general radiance expression (Equation (2)), and the second part is known as the sphere multiplier [32]. The sphere multiplier, denoted as *M*, is a dimensionless quantity that considers the radiance increment resulting from multiple reflections.

(4) $$M=\frac{p}{1-p(1-f)}$$

The value of *M* is influenced by both the sphere surface’s reflectance (*p*) and the port fraction. This port fraction, denoted as *f*, is defined as a function of the input port area (*A_i_*), the output port area (*A_o_*), and the sphere wall area (*A_s_*), and can be expressed as:

(5) $$f=\frac{A_{i\;}+A_{o}}{A_{s}}$$

The design of the integrating sphere aimed to achieve a balance between light distribution and brightness while adhering to established guidelines for the optimal functioning of integrating spheres. In accordance with these guidelines, commercially available integrating spheres typically have diameters ranging from 50 mm to 250 mm with openings that do not exceed 5% of the sphere’s total area and an angle of light incidence not greater than 10 degrees. Following the design guidelines cited before, a 150 mm outer diameter and 140 mm inner diameter sphere was designed. The design of the integrating sphere was realized using computer-aided design software (Autodesk Fusion 360, Autodesk, Inc., San Francisco, CA, USA) and fabricated from white Polylactic Acid (PLA) material using a 3D printer (model Flashforge Guider II, Zhejiang Flashforge 3D Technology Co., Ltd., Jinhua, China) with a filament thickness of 1.75 mm. To enable convenient swapping of various silk cocoons for measurements, the integrating sphere was divided into two components: the upper and bottom sections (Figure 3b). The bottom section was designed to accommodate four LEDs as internal light sources for the integrating sphere, utilizing four 5 mm diameter holes.

The upper section of the integrating sphere featured a cylindrical structure measuring 130 mm in length and 33 mm in diameter. This design was specifically created to securely accommodate the camera lens while also minimizing any potential light interference from external sources. On the other hand, the lower section was equipped with four supports intended for holding silk cocoon samples and the ColorChecker during experiments. These supports were meticulously designed with dimensions of 5 mm in width and 30 mm in length to efficiently preserve the inherent characteristics of the integrating sphere. To ensure stability, grooves measuring 5 mm in width, 5 mm in height, and 3 mm in depth were incorporated at the points where the supports were positioned. Additionally, to minimize light interception, a glass slide measuring 75 mm × 52 mm was employed as the stage and positioned at the corners of the four supports.

To achieve uniform light distribution, a coating process was performed. A mixture of white paint (model PXI453901/4L, NOROO Paint and Coatings, Anyang, Republic of Korea) and distilled water at a 5:1 ratio was prepared. The mixture was stirred and allowed to settle to achieve a uniform blend [30]. The interior of the integrating sphere was coated with the prepared mixture of white paint and water in three layers, each having a designated drying time of 10 hours. This meticulous process was undertaken to ensure a uniform and consistent paint coating on the inner walls of the integrating sphere (see Figure 3c). The reflectance spectrum of the white paint and distilled water mixture was measured and compared to a Spectralon white standard (model USRS-99-010, Labsphere Inc., North Sutton, NH, USA) using an integrating sphere (model 2P4/M, Thorlabs, Newton, NJ, USA) and a compact CCD spectrometer (model CCS200/M, Thorlabs, Newton, NJ, USA). The results demonstrated that white paint reflects 93% of the light (see Figure 3d), which is sufficient for multiple scattering purposes. The high reflectivity of white paint value validates its suitability for use as the reflecting material in the integrating sphere for efficient light reflection and scattering within the integrating sphere. These unique properties make the integrating sphere particularly suitable for measuring the absolute color of a silk cocoon by providing illumination from all directions.

#### 2.1.2. Light Emitting Diode

For the light source within the integrating sphere, we utilized a white LED with a wavelength ranging from 420 nm to 700 nm, as depicted in Figure 3e. To ensure effective diffusion of light throughout the interior space of the integrating sphere, following geometric optics principles within the integrating sphere [33,34], we chose a white LED with a broader wavelength range. To achieve uniform illumination, four LEDs were precisely positioned in the designated holes in the lower section of the integrating sphere and interconnected through soldering. These LEDs were angled in a way that the emitted light was directed toward the surrounding walls of the integrating sphere instead of directly illuminating the stage. This configuration enabled optimal engagement of light with the internal surface of the integrating sphere. As a result, the light circulating within the integrating sphere effectively traverses the entire internal surface, resulting in a uniform distribution of irradiance. The LED connections were configured in series, powered by an applied voltage of 2.7 V. Upon activation, the emitted light from the LEDs was uniformly distributed and radiated in all directions after multiple scattering and reflection events (see Figure 3f).

#### 2.1.3. Silk Cocoon Sample Preparation

In practical settings, silk cocoons are commonly categorized into three distinct colors visible to the human eye: apricot, light green, and yellow. For our study, we specifically focused on these color categories. To streamline our experimental procedure, we meticulously prepared silk cocoons representing each color category. This was achieved by employing a 1 cm diameter punch to precisely cut them into uniform pieces, facilitating their seamless placement within the integrating sphere alongside the ColorChecker.

#### 2.1.4. Imaging Setup and Configuration

A color camera (model GS3-U3-50S5C-c 2/3″ FLIR Grasshopper 3, Teledyne FLIR LLC, Wilsonville, OR, USA) consisting of a 2/3 inches sensor with a maximum resolution of 2448 × 2048 square pixels of length 5.3 µm, was strategically positioned on a designated mount within the upper section of the integrating sphere. This placement was critical to ensure optimal focusing and illumination for the stage area where we placed our silk cocoon samples. The camera was connected to a PC via a USB cable and utilized a manually adjusted C-mount zoom lens (model 87-536 0.15X-0.5X Non-Telecentric Lens, Edmund Optics, Barrington, NJ, USA) with a focal length range of 50–179.8 mm and an aperture that could be adjusted from f/2.8 to f/22. We have used FLIR FlyCapture 2.0 software to capture the images. To ensure the highest image quality, we chose to save the images in the TIFF format, which is known for preserving fine details without loss during storage.

To achieve optimal results, we set specific illumination levels by defining saturation limits for both the silk cocoon samples and the ColorChecker. Since each silk cocoon sample (apricot, light green, and yellow) had distinct color characteristics, we needed tailored illumination conditions for each. Consequently, we manually adjusted various camera settings, including brightness, shutter speed, gain, frame rate, and white balance (red channel) for each silk cocoon sample to ensure the most accurate color measurement.

#### 2.1.5. Experimental Setup

**Figure 4:**
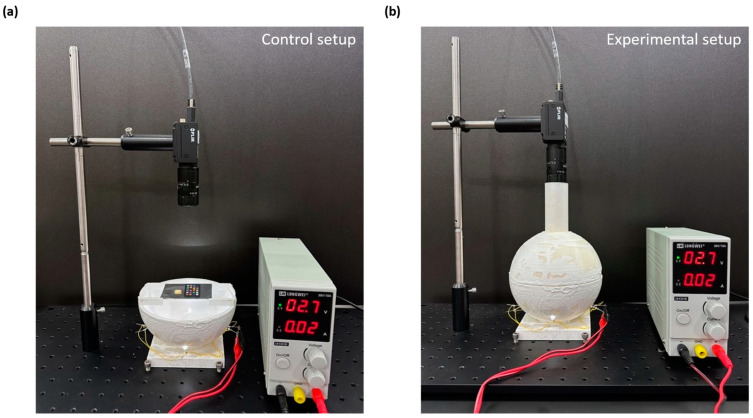
Experimental setup: (**a**) Depicts the setup for the control group where the silk cocoon sample and ColorChecker are positioned on a sample holding stage within the lower section of the integrating sphere while the upper section remains open, exposing the sample to indoor lighting; (**b**) The integrating sphere system is shown with the upper section attached to the lower section, and LEDs turned on to ensure uniform illumination inside the sphere. This setup uniformly illuminates both the silk cocoon sample and the enclosed ColorChecker, facilitating data capture for the treatment group.

Our experimental approach involved the utilization of two distinct setups: the control setup (Figure 4a) and the experimental setup (Figure 4b), each contributing uniquely to our research. Our workflow can be succinctly summarized as follows: We began by meticulously preparing the silk cocoon samples, employing a 1 cm diameter punch to create uniform pieces. These carefully prepared samples were then positioned on the stage inside the integrating sphere, alongside the ColorChecker (Figure 4a). In this configuration, we used the upper section of the integrating sphere open to capture the images of the control group, serving as our baseline reference. After successfully acquiring the control group images, we securely placed the upper section of the integrating sphere to capture experimental group images. We employed a high-performance color camera, equipped with a telecentric lens to capture images of the arranged samples under uniform illumination conditions (Figure 4b). To acquire these images, we used the FLIR FlyCapture software, which provided comprehensive data for subsequent analysis. Following image acquisition, we performed a crucial step: color calibration. The color calibration was performed using MATLAB (Version R2022b, MathWorks, Natick, MA, USA), incorporating a power function to ensure the precision and accuracy of absolute color measurements of silk cocoons featuring corrugated microstructures.

#### 2.1.6. ColorChecker

**Figure 5:**
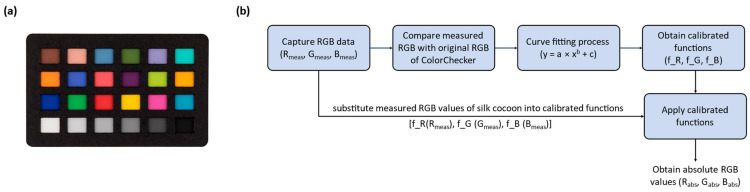
RGB color calibration through curve fitting: (**a**) Calibrite ColorChecker Classic-Nano. The ColorChecker Classic-Nano, measuring 24 mm by 40 mm, was well-suited for alignment with the field of view (FOV) of our camera and integrating sphere setup; (**b**) Flow diagram of the color calibration process. RGB color calibration of photographed ColorChecker using curve fitting in MATLAB (R2022b) to align measured RGB (R_meas_, G_meas_, B_meas_) values with actual ColorChecker RGB (R_orig_, G_orig_, B_orig_) values, enabling absolute color measurement of silk cocoon within the integrating sphere setup.

To measure the absolute color of silk cocoons, we utilized the calibrated ColorChecker Classic-Nano, which is the smallest version (24 × 40 mm) in the ColorChecker series (Figure 5a). The ColorChecker Nano comprises 24 distinct colors: dark skin, light skin, blue sky, foliage, blue flower, bluish green, orange, purple red, moderate red, purple, yellow green, orange yellow, blue, green, red, yellow, magenta, cyan, white, neutral 8, neutral 6.5, neutral 5, neutral 3.5, and black [35]. It is important to mention that the ColorChecker is opaque, whereas silk cocoons possess a degree of translucency, allowing light from the bottom of the integrating sphere to pass through. Consequently, this difference results in varying light intensities hitting the ColorChecker depending on the transparency of the materials placed inside. To ensure consistent and accurate measurement of light intensity on the ColorChecker, regardless of the presence of silk cocoons, we covered the glass slide with highly reflective aluminum foil. This step ensured that our measurements remained unaffected by the variable transparency of the silk cocoons, allowing us to maintain the integrity of our color measurements.

### 2.2. Methods

#### Color Calibration

Color calibration typically falls into two primary categories: device-based and image-based methods. Device-based approaches, extensively studied in prior research [36,37], aim to enhance camera sensors’ ability to adjust autonomously to varying color values influenced by different lighting conditions in a scene. On the other hand, image-based methods serve to counteract color distortion stemming from alterations in lighting or changes in camera sensor responses. Particularly prevalent in professional photography, this method ensures the preservation of consistent color tones. It achieves this by utilizing standard color calibration charts to linearly align pixel values from the image with the standardized color values of the calibration chart [38]. Previous studies emphasize color calibration as one of the most important factors to ensure color measurement accuracy and minimize error [39,40]. In a study by researchers in [41], precise RGB spectral response functions were predicted using ColorChecker, demonstrating minimal root mean square error between the actual values and their recovered RGB spectral response, all achieved through color calibration. Sunoj et al. [38] devised a method using the standard ColorChecker. This method demonstrated that employing red (R), green (G), and blue (B) patches yielded comparable performance to 24-color patches, proving its simplicity and practicality. This emphasizes the importance of standardized color calibration techniques in achieving accurate and reliable color measurements across various applications. Through the color calibration process, a correlation is established between the image’s color values and those standardized values, enabling the application of these findings for color standardization and calibration. Despite the availability of diverse commercial calibration charts containing varying quantities of color patches, ranging from as few as six to several hundred, the 24-color patch calibration chart remains widely favored and employed across multiple applications.

The RGB values of the ColorChecker inside the integrating sphere system, which depend on the intensity of illumination, exhibit deviations from their original RGB values. Therefore, it is essential to calibrate the RGB values obtained from the photographed ColorChecker to align them with the actual RGB values of the ColorChecker [42]. This calibration process involves taking the RGB values from the photographed ColorChecker (R_meas_, G_meas_, B_meas_) and correcting them by comparing them with the original ColorChecker RGB (R_orig_, G_orig_, B_orig_) values (refer to Figure 5b). We began by measuring the RGB values (R_meas_, G_meas_, B_meas_) of the ColorChecker using the camera. Next, we plotted the measured RGB (R_meas_, G_meas_, B_meas_) values against the original RGB (R_orig_, G_orig_, B_orig_) values of the ColorChecker to create a calibration curve. This helped establish the relationship between the measured and original RGB values. We employed MATLAB R2022b’s curve fitting tool to fit a power function model to the data points obtained from the ColorChecker measurements.

The curve fitting procedure utilized a power function model, conventionally represented as:

*y* = *a* × *x^b^* (6)

In this equation, ‘*a*’ and ‘*b*’ are parameters determined through the curve fitting process, where ‘*x*’ represents the independent variable and ‘*y*’ represents the dependent variable. The function effectively transforms the input value ‘*x*’ by raising it to the power ‘*b*’ and scaling the result by the factor ‘*a*’ to obtain the output value. The power function was chosen for its versatility in modeling nonlinear relationships and handling various data distributions and patterns [43]. Moreover, it offers the added advantage of computational efficiency due to its relative simplicity [44].

For a more precise modeling approach, a constant term ‘*c*’ was introduced to the power function, resulting in the following model:

*y* = *a* × *x^b^* + *c* (7)

The constant term ‘*c*’, determined through the curve fitting process, adjusts the linear offset between ‘*x*’ and ‘*y*’. The resultant curve-fitting data for each sample are presented in detail in Section 3. Once the power function model was fitted to the data, we used the resulting functions (f_R, f_G, f_B) to calibrate the RGB values of the ColorChecker. The calibrated functions for the RGB channels (f_R, f_G, f_B) are summarized in Table 1, Table 2 and Table 3. Finally, after substituting the measured raw RGB values (R_meas_, G_meas_, B_meas_) of the silk cocoons into the calibrated functions (f_R, f_G, f_B), we determined the absolute RGB values (R_abs_, G_abs_, B_abs_) of the silk cocoons exhibiting a corrugated microstructure.

## 3. Results

**Figure 6:**
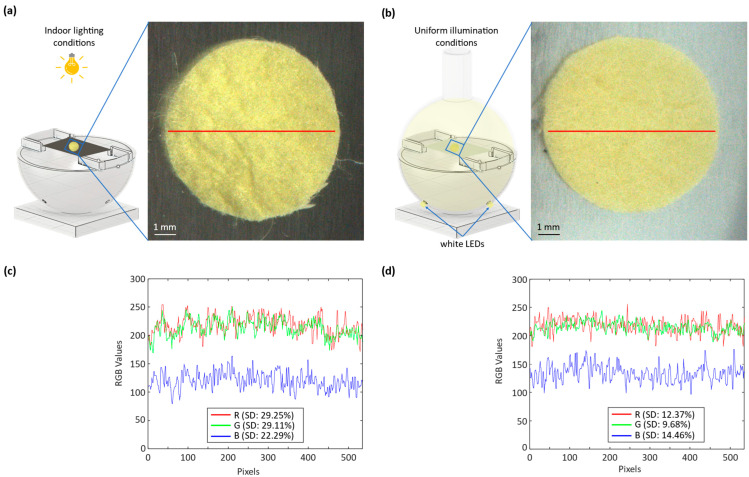
Characterization of illumination conditions and color analysis of silk cocoon samples: (**a**) Silk cocoon sample captured under indoor light illumination; (**b**) A silk cocoon sample is shown placed inside an integrating sphere with the image captured under uniform illumination. The red line in (**a**) and (**b**) delineates the region used for calculating the standard deviation and the RGB intensity profile; (**c**) RGB intensity profile, along with its standard deviation, for the pictures taken under indoor lighting conditions; (**d**) RGB intensity profile, along with its standard deviation, for the pictures taken under uniform illumination inside the integrating sphere.

Figure 6 presents a thorough comparative analysis of the silk cocoon samples under different illumination conditions. In Figure 6a, we observe an image of the silk cocoon sample under indoor lighting, revealing how indoor illumination affects its visual characteristics. Figure 6b shows the same silk cocoon sample placed within an integrating sphere, ensuring uniform illumination. These images allow for a visual comparison of the sample’s color and texture under different lighting conditions. Figure 6c provides further insights by presenting the intensity profiles of the red–green–blue (RGB) channels for images taken under indoor lighting conditions. The corresponding standard deviations for each channel are reported as R (SD—29.25%), G (SD—29.11%), and B (SD—22.29%). These standard deviations offer a measure of the variations in color intensity within the captured images, indicating the sensitivity of the sample’s appearance to indoor lighting conditions. In contrast, Figure 6d displays the intensity profiles of the RGB channels for images captured under uniform illumination inside the integrating sphere. Here, the standard deviations for each channel are reported as R (SD—12.37%), G (SD—9.68%), and B (SD—14.46%). The significantly reduced standard deviations indicate that the uniform illumination provided by the integrating sphere results in a more consistent color representation of the silk cocoon sample.

This comparison substantiates the efficacy of the devised optical system with the integrating sphere. The images captured under uniform illumination exhibit a smooth surface texture, minimizing the visible hills and valleys compared to the images captured under uneven illumination conditions. It highlights how the uniform illumination provided by the system minimizes color variations caused by uneven lighting, thereby ensuring a more accurate and consistent extraction of absolute RGB values from silk cocoon samples. Ultimately, this finding not only aids in understanding the intricate colors of silk cocoons but also presents an innovative approach applicable to various specimens with uneven surface textures.

The color profiles of the silk cocoon samples under indoor lighting exhibited significant variability, primarily attributed to the uneven surface texture of the cocoons and the viewing angle. This observation underscores the challenges in accurately assessing the colors of silk cocoons. When we captured the samples under indoor lighting conditions and extracted their RGB values using MATLAB R2022b, we observed that the extracted RGB values appeared darker than expected. We attributed this discrepancy to the shadowing effects caused by the wrinkled surface of the silk cocoons, featuring corrugated microstructures, resulting in color distortion.

To determine the absolute colors of silk cocoon samples, we employed our integrating sphere imaging setup. The samples were illuminated inside the integrating sphere and placed alongside the ColorChecker, using an array of white LEDs operating at 2.7 V. Subsequently, we extracted the RGB values from the photographed ColorChecker (R_meas_, G_meas_, B_meas_) and compared them with the original RGB values of the ColorChecker (R_orig_, G_orig_, B_orig_). For the analysis, we utilized a curve-fitting process with a Power function to calibrate the RGB functions (f_R, f_G, f_B). The results of this curve-fitting procedure for our three sample sets demonstrate a linear relationship between the R, G, and B values.

By substituting the measured raw RGB values of the silk cocoons into the calibrated functions (f_R, f_G, f_B), we calculated the absolute RGB values (R_abs_, G_abs_, B_abs_) of silk cocoons. The resulting graph showed a linear relationship between the RGB values and the corresponding colors of the silk cocoon samples within the integrating sphere setup. Notably, the calibrated RGB values obtained from the Power function fitting curve exhibited a smooth transition and closely matched the expected values of the ColorChecker. This agreement serves to validate the effectiveness of our calibration procedure and strengthens the credibility of the absolute RGB values of the silk cocoon samples we obtained.

**Figure 7:**
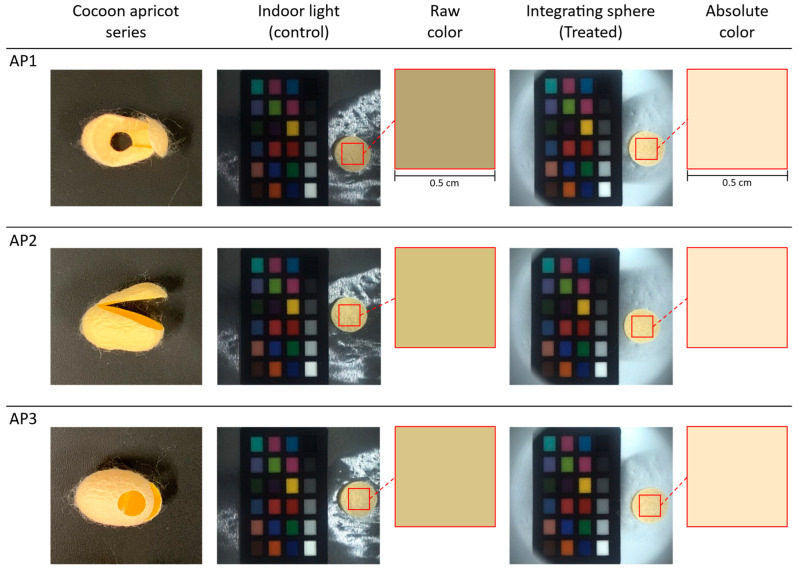
Color analysis and uniform illumination effects on apricot silk cocoon samples. Three different silk cocoon samples in the apricot color series were captured alongside the ColorChecker under indoor lighting conditions (control group) and inside of integrating sphere (treated group). A region of interest (ROI) measuring 0.5 cm × 0.5 cm is selected. Raw color extracted from control group images (left) exhibits color variations due to the shadowing effects arising from the corrugated structure of the silk cocoon’s surface. Treatment group images (right) display the absolute colors of the silk cocoons obtained after color calibration, indicating the successful mitigation of shadowing effects caused by the uneven surface texture of apricot-colored silk cocoons.

The three silk cocoon samples in the apricot color series were labeled as AP1, AP2, and AP3. We captured images of the entire silk cocoon from the control group with the upper section of the integrating sphere open, allowing indoor lighting to illuminate the samples (see Figure 7). These lighting conditions revealed subtle variations in the apricot-colored silk cocoons, influenced by changes in light intensities, incident light angles, and viewing perspectives. In contrast, the treatment group images were taken with the upper section of the integrating sphere closed, resulting in uniform light illumination from all directions. Notably, the raw colors extracted from the control group appeared relatively darker, a phenomenon attributed to the shadowing effects caused by the silk cocoon’s uneven and corrugated surface. However, colors extracted from images captured under uniform illumination using a color calibration procedure showed absolute representative colors of silk cocoons consistent across all three apricot samples. This observation suggests that our customized integrating sphere setup, providing uniform illumination, effectively addressed the physical limitations associated with the uneven surface texture of the silk cocoons featuring a corrugated microstructure.

The camera settings for these captures were configured as follows: exposure 1.2 EV, sharpness 1024, shutter 66 ms, gain 8.868, white balance (red) 669, white balance (blue) 771, and gamma OFF. During raw data measurement and absolute color calculation, we defined a region of interest (ROI) measuring 0.5 cm × 0.5 cm from the silk cocoon sample.

**Figure 8:**
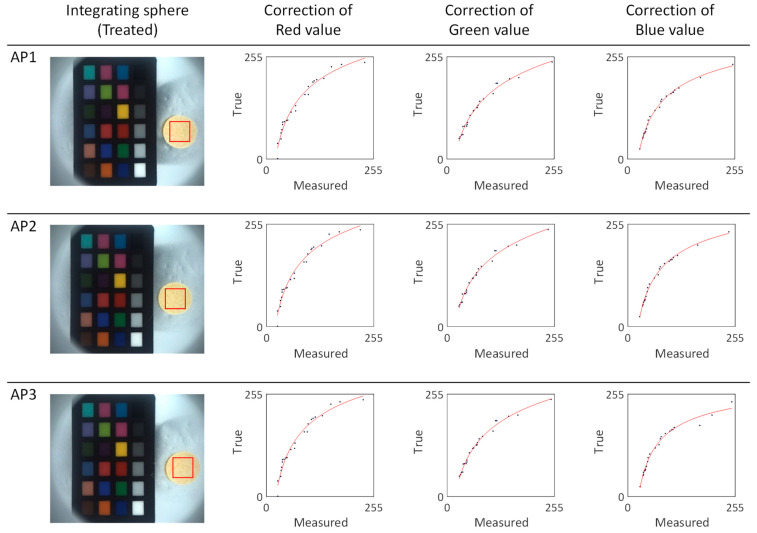
Color calibration and correction of the apricot silk cocoon series. The red line depicts the fitted power function graphs derived from the apricot series of silk cocoon samples, which were captured within the integrated sphere setup along with the ColorChecker. The blue dots on the graph represent a comparative analysis between the measured colors of the ColorChecker within the integrating sphere and the inherent colors of the original ColorChecker.

To determine the absolute color of the apricot series silk cocoons shown in Figure 7, we subdivided the region of interest (ROI) of the measured silk cocoon samples and extracted the RGB values. These RGB values were then input into the calibrated power functions (f_R, f_G, f_B) represented by the red lines (Figure 8). The calibrated function for each sample in the apricot series of silk cocoons is summarized in Table 1. The obtained absolute color of the silk cocoons, represented as the corrected RGB values for each sample in the apricot series, is as follows: AP1 = R: 251, G: 232, B: 200; AP2 = R: 254, G: 234, B: 200; AP3 = R: 251, G: 231, B: 195.

**Figure 9:**
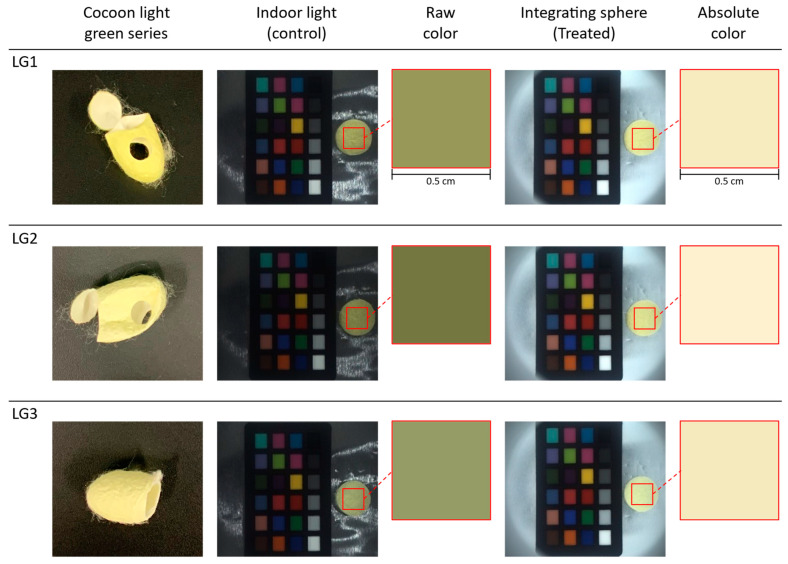
Color analysis and uniform illumination effects on light green series silk cocoon samples. Three different silk cocoon samples in the light green series were captured together with ColorChecker under indoor lighting conditions (control group) and inside of integrating sphere setup (treated group). An ROI measuring 0.5 cm × 0.5 cm was selected. Raw color extracted from control group images (left) displays variations caused by shadowing effects resulting from the uneven silk cocoon’s surface. In contrast, the treatment group images (right) exhibit the absolute colors of the light green silk cocoons after successful color calibration.

The three silk cocoon samples within the light green series of silk cocoons were labeled as LG1, LG2, and LG3. We captured the images of the prepared silk cocoon samples of the control group (see Figure 9). In the control group images, the upper section of the integrating sphere was open, allowing indoor lighting to illuminate the silk cocoon samples. These images unveiled subtle variations among the light green series of silk cocoon samples, influenced by various factors such as differing light intensities, angles of incident light, and viewing perspectives. In contrast, the treated group images were captured with the upper section of the integrating sphere firmly attached to the bottom section, ensuring uniform light exposure from all directions. Notably, the raw colors extracted from the control group appear relatively darker, primarily due to the shadowing effect resulting from the uneven textured surface of the silk cocoon. Conversely, colors extracted from treated images, subjected to uniform illumination, exhibited consistent absolute colors among all three light green silk cocoon samples. This observation suggests that the physical limitations associated with silk cocoons, stemming from their corrugated microstructure, were effectively addressed in the treated group. The camera settings used for these captures were configured as follows: exposure 1.2 EV, sharpness 1024, shutter 66 ms, gain 8.868, white balance (red) 669, white balance (blue) 771, and gamma OFF. In our color analysis, we selected a region of interest (ROI) measuring 0.5 cm × 0.5 cm. This meticulous ROI selection ensured both the accuracy and consistency of our absolute color measurement across all samples of the light green series of silk cocoons.

**Figure 10:**
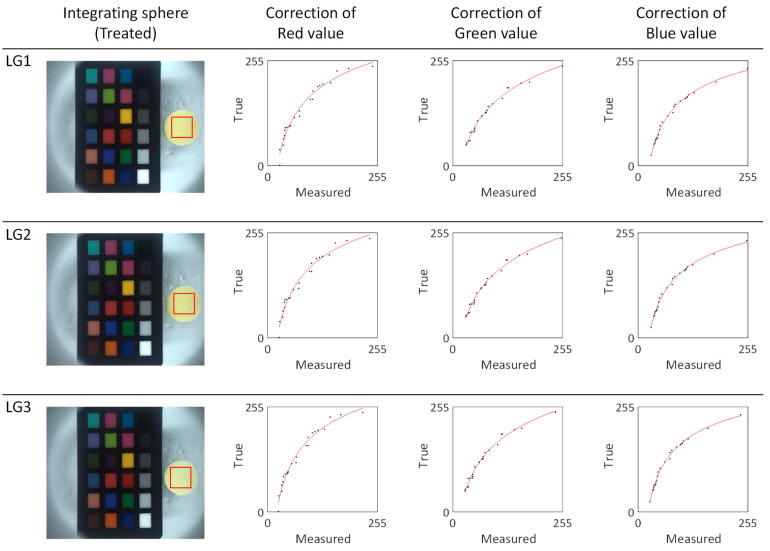
Color calibration and correction of the light green silk cocoon series. The red line represents the fitted power function graphs derived from the light green series of silk cocoon samples captured within the integrating sphere setup, along with the ColorChecker, and their corresponding corrected RGB values. The blue dots on the graph indicate a comparative analysis between the measured colors of the ColorChecker within the integrating sphere and the inherent colors of the original ColorChecker. These points were curve-fitted using a power function, which is represented by the red line in the graph.

We proceeded to perform a calibration of the RGB values and calculation of the absolute color of the light green series silk cocoons, as shown in Figure 10. We chose a 0.5 cm × 0.5 cm region of interest (ROI) and extracted RGB values from these ROIs. These RGB values were then input into the calibrated power functions (f_R, f_G, f_B) represented by the red line (see Figure 10). The calibrated function for each sample in the light green series is summarized in Table 2. The resulting absolute colors for the light green silk cocoons are presented as the corrected RGB values for each sample, as follows: LG1 = R: 244, G: 234, B: 193. LG2 = R: 250, G: 241, B: 209. LG3 = R: 242, G: 232, B: 192.

**Figure 11:**
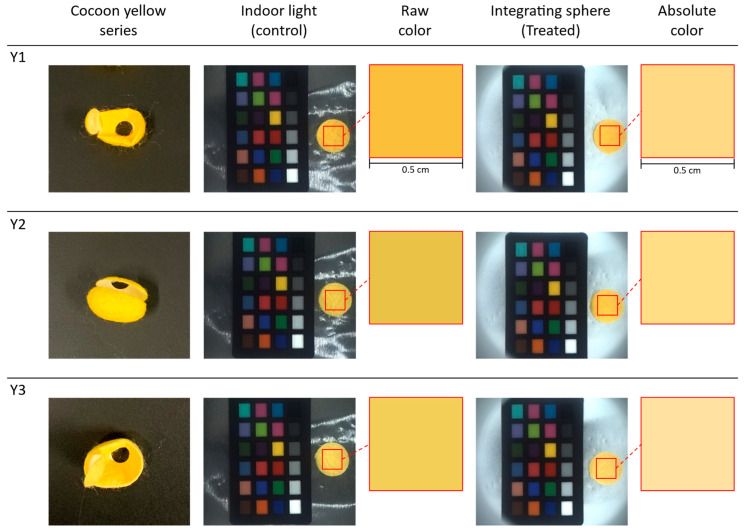
Color analysis and uniform illumination effects on yellow silk cocoon samples. Three different silk cocoon samples in the yellow series were captured together with ColorChecker under two distinct lighting conditions: indoor lighting (control group) and inside an integrating sphere (treated group). An ROI measuring 0.5 cm × 0.5 cm is selected for analysis. The raw colors extracted from the control group images (left) reveal variations attributed to shadowing effects caused by the uneven surface of silk cocoons. In contrast, the images from the treatment group (right) exhibit the absolute colors of the yellow-colored silk cocoons following successful color calibration.

The three silk cocoon samples within the yellow series were labeled as Y1, Y2, and Y3. We captured control group images under indoor lighting conditions. Color variations became noticeable in the images of the yellow color series of silk cocoon samples and became apparent under varying lighting intensities, incident light angles, and viewing perspectives (see Figure 11). The raw color extracted from the control group appeared darker, attributed to the shadowing caused by the corrugated microstructure of the silk cocoon. On the other hand, the treated group images were acquired with the upper section of the integrating sphere attached to the bottom section, ensuring uniform light illumination from all directions. The images taken under uniform illumination exhibited smoother surface characteristics, indicating that physical limitations associated with the uneven silk cocoon surface were addressed in the treated group.

Our camera settings for these captures included exposure at 1.2 EV, sharpness at 1024, shutter speed of 66 ms, gain set at 8.868, white balance (red) adjusted to 669, white balance (blue) at 771, and gamma turned OFF. During the measurement of raw RGB values and calculation of the absolute colors, we defined a 0.5 cm × 0.5 cm region of interest (ROI).

**Figure 12:**
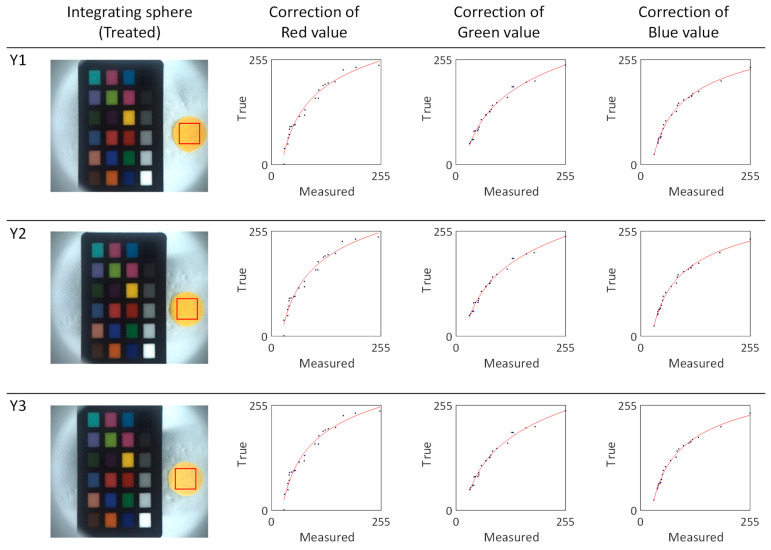
Color calibration and correction of the yellow silk cocoon series. The red line depicts the fitted power function graphs derived from the yellow series of silk cocoon samples captured within the integrating sphere setup, along with the ColorChecker, and their corresponding corrected RGB values. The blue dots represent a comparative analysis between the measured colors of the ColorChecker within the integrating sphere and the inherent colors of the original ColorChecker. These points were curve-fitted using a power function, as shown by the red line in the graph above.

To determine the absolute colors of the yellow silk cocoons, we selected the region of interest (ROI) measuring 0.5 cm × 0.5 cm from the measured silk cocoon samples. From this ROI, we extracted the RGB values and applied calibrated power functions (f_R, f_G, f_B) represented by the red lines (see Figure 12). The calibrated function for each sample in the yellow series silk of cocoons is summarized in Table 3. The absolute colors of silk cocoons, presented as the corrected RGB values for each sample, are as follows: Y1 = R: 247, G: 216, B: 137. Y2 = R: 247, G: 220, B: 138. Y3 = R: 247, G: 224, B: 164.

## 4. Discussion

The main objective of this study was to address the challenges posed by the intricate surface texture of the silk cocoons, which often resulted in inaccuracies when capturing their true RGB values. Notably, when silk cocoon images were taken under conventional indoor lighting conditions (as shown in Figure 8, Figure 10, and Figure 12), a substantial disparity was observed between the RGB values recorded from the photographed images and the actual colors. This disparity was attributed to the complex wrinkled surface of silk cocoons with corrugated microstructures, making precise color measurements difficult. Additionally, real-world observations revealed that color variations were influenced by factors such as differing light intensities, angles of incident light, and viewing perspectives.

To overcome these challenges, a computational approach was devised to extract the absolute colors of silk cocoons. To minimize color distortion caused by the uneven surface of the silk cocoon’s corrugated microstructure, a customized integrating sphere system was designed and 3D printed. The inner walls of the sphere were coated with a mixture of commercially available white paint and water to maximize light reflection. As a result, the customized integrating sphere system provided uniform light distribution and irradiation from all angles by enabling multiple scattering and reflection of incident light.

The core of this study focused on the calibration process, which involved calibrating the colors of the ColorChecker images captured within the integrating sphere system using MATLAB R2022b. It is noteworthy that the RGB values within the integrating sphere setup deviated from the original ColorChecker’s true RGB values due to varying imaging conditions, such as illumination intensity. Therefore, the measured RGB values of the ColorChecker (R_meas_, G_meas_, B_meas_) within the integrating sphere setup were compared with the original RGB values of the ColorChecker (R_orig_, G_orig_, B_orig_) to assess deviations. A power function was applied using MATLAB 2022b’s curve fitting module to align the original and measured ColorChecker RGB values. The enhanced power function model was used to adjust the linear offset between ‘*x*’ and ‘*y*’. This curve-fitting process resulted in the calibration of the RGB functions (f_R, f_G, f_B). This calibration effectively linearized the RGB values, as demonstrated in Figure 9, Figure 11, and Figure 12. This linearization enabled the extraction of absolute RGB values under specific imaging conditions. Furthermore, the silk cocoons captured within the integrating sphere setup exhibited a smoother and more linear surface compared to those captured under conventional indoor lighting conditions.

This study effectively employed a computational approach to mitigate color variation issues associated with silk cocoons’ corrugated microstructure. The newly developed system significantly streamlines color determination and eliminates the inaccuracies caused by shadowing and viewing angle distortions. By developing a more precise method for measuring the absolute color of silk cocoons, this research could greatly impact both the textile industry and the agriculture sector. In the textile industry, improved color measurement techniques could lead to more efficient breeding and extraction processes, reducing waste and increasing product quality. In medical fields, enhancing the accuracy of silk cocoon color measurement could minimize experimental errors, thereby increasing the reliability of applications such as biosensing and bioimaging. Additionally, by providing a standardized method for color measurement, this research may facilitate comparison and collaboration between different research efforts, advancing the overall field of silk cocoon utilization. We hope that this work will contribute to the more effective and wide-ranging use of this remarkable biomaterial.

### Comparative Assessment of the Developed System to Existing Systems

In the pursuit of accurate color assessment for silk cocoons, various methodologies have been explored, each having distinct strengths and limitations. One widely adopted approach involves the use of UV–visible spectroscopy, as demonstrated by Cheng, L., et al. [17,45]. While this method attains high accuracy in color assessment, its prohibitively expensive nature poses a significant barrier, limiting its practical application on a broader scale. An alternative avenue explored in the literature involves digital camera-based techniques, as evidenced by several studies [8,18,19,20]. These techniques leverage digital images for color analysis, offering a more accessible option. However, a notable drawback lies in the reliance on the proportions of RGB values without adequate color calibration. This limitation becomes a focal point in our discussion, as it impedes the measurement of absolute color, a challenge effectively addressed by our proposed method.

Also, an integrating sphere has been utilized in conjunction with a colorimeter to obtain color index measurements for silk cocoons [21]. Despite the valuable insights gained, the use of a white cocoon as a control introduces complexity in assessing the absolute color of silk cocoons. This limitation underscores the need for methodologies that overcome such challenges, paving the way for more comprehensive color evaluation. Another approach centers on the RGB color component difference for silk cocoon color identification [16]. However, this method is not without its limitations. Issues arise from the reliance on data peak density and assumptions about ambient light conditions, aspects that our proposed technique successfully addresses through the incorporation of absolute color measurement. Visible spectroscopy, as employed by Kim et al. in [23], to measure the yellowish index of non-woven silk fabrics in CIE 1931 color space. However, the high cost and demanding experimental conditions associated with the utilization of a spectrophotometer, render it impractical for applications such as small- to medium-sized silkworm breeding and sorting processes. This limitation emphasizes the necessity for more cost-effective user-friendly alternatives (see Table 4).

Our developed technique introduces an optical system utilizing an integrating sphere, ensuring consistent and uniform illumination from all orientations. The calibration of RGB values using a ColorChecker enhances precision, facilitating the accurate extraction of absolute RGB values for absolute color measurement. Importantly, our method stands out for its cost-effectiveness, making it a practical choice for various applications. Furthermore, the in-house fabrication of the integrating sphere allowed for customization, enabling tailored sample staging and adjustments to the light input port at a reduced cost. This adaptability enhances the versatility of our technique, making it applicable to a broader range of scenarios.

While the use of an integrating sphere, which is traditionally associated with absolute measurements, might seem at odds with the comparative aspect introduced by employing a ColorChecker, it is important to note its significance. Integrating spheres are renowned for their precision through uniform illumination and total light collection, typically associated with absolute measurement techniques. However, when dealing with materials like silk cocoons characterized by their corrugated microstructure, this requires adopting a color calibration process, which differs from the usual procedure. This calibration is not a limitation but a vital step in ensuring accuracy and comparability amidst the inherent surface variability of materials like silk cocoons. By establishing a calibrated reference using ColorChecker, we can effectively address material-specific nuances, enabling consistent and reliable color measurements across diverse samples and varied conditions.

## 5. Conclusions

In this study, we addressed the challenges associated with accurately assessing the absolute colors of silk cocoons. These challenges primarily arise from their textured surface, characterized by corrugated microstructures, and their sensitivity to lighting conditions during color measurement. We developed a custom optical system using an integrating sphere. This integrating sphere provided uniform illumination to the silk cocoon samples from all geometrical orientations. Comparing our method to conventional indoor lighting conditions, we found that images captured under uniform illuminations had lower standard deviations (SD) in their RGB profiles. Next, we implemented a color calibration process utilizing the curve-fitting capabilities of MATLAB. We conducted experiments with apricot, light green, and yellow silk cocoon samples. We found that images captured under indoor lighting conditions displayed significant variations among the three different samples of each silk cocoon color. Conversely, colors extracted from uniform illumination using an integrating sphere accurately represented the absolute colors of silk cocoons and demonstrated consistency across all three samples of each color group.

This approach has led to more precise and consistent color measurements of silk cocoons. The implications of this study extend to the textile industry, agriculture, and medical applications, where it has the potential to enhance breeding practices, product quality, and the reliability of technologies built on silk cocoon materials. Our methodology establishes a solid foundation for future applications in accurately determining color properties of similar materials, which could be invaluable across various scientific and industrial domains. Further research could investigate how this method can perform under varying lighting conditions and with various camera types, providing a more comprehensive understanding of the current setup’s limitations and thereby contributing to its refinement.

## Figures and Tables

**Table 1:** Calibrated functions for apricot series of silk cocoons.

Sample	Calibrated Functions		
	f_R	f_G	f_B
AP1	−1226 × *x*^−0.218^ + 624.5	−2410 × *x*^−0.0475^ + 2098	−1270 × *x*^−0.3454^ + 420.8
AP2	−1211 × *x*^−0.2225^ + 614.7	−1842 × *x*^−0.06702^ + 1519	−1323 × *x*^−0.3752^ + 401.2
AP3	−1228 × *x*^−0.2613^ + 547.2	−1437 × *x*^−0.1005^ + 1069	−1713 × *x*^−0.5245^ + 315

**Table 2:** Calibrated functions for a light green series of silk cocoons.

Sample	Calibrated Functions		
	f_R	f_G	f_B
LG1	−1242 × *x*^−0.1975^ + 670.6	(−3.595 × 10^4^) × *x*^−0.00262^ + (3.568 × 10^4^)	−1211 × *x*^−0.295^ + 468.2
LG2	−1232 × *x*^−0.2057^ + 650	(−3.663 × 10^4^) × *x*^−0.00252^ + (3.636 × 10^4^)	−1234 × *x*^−0.3197^ + 442.2
LG3	−1238 × *x*^−0.205^ + 661.4	−3468 × *x*^−0.03035^ + 3197	−1202 × *x*^−0.3151^ + 447.8

**Table 3:** Calibrated functions for yellow series of silk cocoons.

Sample	Calibrated Functions		
	f_R	f_G	f_B
Y1	−1260 × *x*^−0.2084^ + 649.1	8518 × *x*^0.01054^ − 8788	−1230 × *x*^−0.2903^ + 477.1
Y2	−1275 × *x*^−0.1799^ + 723.3	3490 × *x*^0.02411^ + 3746	−1228 × *x*^−0.2967^ + 468
Y3	−1248 × *x*^−0.2157^ + 629	(−2.311 × 10^5^) × *x*^−0.00040^ + (2.308 × 10^5^)	−1230 × *x*^−0.2937^ + 472.3

**Table 4:** Noteworthy techniques for silk cocoon color analysis.

Method	Measurement Type	Cost ($)	Reference
UV–visible spectroscopy	Spectrum analysis	2800	[21]
Visible spectroscopy	Spectrum analysis	10,000	[23]
Colorimeter	Color index	12,000	[17,45]
UV–Vis measurement and chemical treatment	Color estimation	5000~50,000	[16]
Our proposed system	Absolute color measurement	1000	

## Data Availability

The data presented in this study are available from the corresponding authors upon request.
